# ImmuSort, a database on gene plasticity and electronic sorting for immune cells

**DOI:** 10.1038/srep10370

**Published:** 2015-05-19

**Authors:** Pingzhang Wang, Yehong Yang, Wenling Han, Dalong Ma

**Affiliations:** 1 Department of Immunology, School of Basic Medical Sciences, Peking University Health Science Center. Key Laboratory of Medical Immunology, Ministry of Health, China. Peking University Center for Human Disease Genomics. No. 38 Xueyuan Road, Beijing, 100191, China; 2 Chinese National Human Genome Center at Beijing. No. 3-707 North YongChang Road, BDA, Beijing, 100176, China; 3 Tip-Science(Beijing) Bioinformatics Co. Ltd. The current affiliation: Institute of Basic Medical Sciences Chinese Academy of Medical Sciences. No. 5 Dong Dan San Tiao, Beijing, 10005, China

## Abstract

Gene expression is highly dynamic and plastic. We present a new immunological database, ImmuSort. Unlike other gene expression databases, ImmuSort provides a convenient way to view global differential gene expression data across thousands of experimental conditions in immune cells. It enables electronic sorting, which is a bioinformatics process to retrieve cell states associated with specific experimental conditions that are mainly based on gene expression intensity. A comparison of gene expression profiles reveals other applications, such as the evaluation of immune cell biomarkers and cell subsets, identification of cell specific and/or disease-associated genes or transcripts, comparison of gene expression in different transcript variants and probe set quality evaluation. A plasticity score is introduced to measure gene plasticity. Average rank and marker evaluation scores are used to evaluate biomarkers. The current version includes 31 human and 17 mouse immune cell groups, comprising 10,422 and 3,929 microarrays derived from public databases, respectively. A total of 20,283 human and 20,963 mouse genes are available to query in the database. Examples show the distinct advantages of the database. The database URL is http://202.85.212.211/Account/ImmuSort.html.

Gene expression profile (GEP) analysis is generally a necessary and important step for a functional gene study, especially for those of genes with unknown functions. Many tools and databases that are coupled with accumulating high-throughput data have been developed for GEP analysis. It has been revealed that gene expression is highly heterogeneous across various tissues and cells, and often changes during the course of development or under different experimental treatments. Thus, the identification of differentially expressed genes at various biological states has been widely conducted to reveal the potential mechanisms involved in cellular processes. Microarray and next-generation sequencing^1^,^2^ represent two technologies that are widely used to detect mRNA levels and differential expression. Many gene expression databases based on these high-throughput technologies^3^,^4^ have been built to provide important functional clues for experimental design and gene function validation. However, the limited experiment conditions and cell states in these databases restricts their usefulness in illustrating the highly dynamic and plastic characteristics of gene expression.

The mammalian immune system is composed of dozens of immune cell groups. GEP analysis plays a vital role in dissecting the function of each cell group. Several immune-cell-related databases exist, such as RefDIC^5^, BloodExpress^6^, ImmGen^7^ and HemaExplorer^8^. Some immune cell and tissue GEP data are included in other general databases, such as BioGPS^9^. However, these databases usually provide a static presentation of expression data from very limited conditions and thus cannot reflect gene regulation properties under a wide range of factors, such as intra- or extra-cellular stimuli, which can greatly modify gene expression levels. In most wet experiments conducted during gene function studies, the focus is the treatment conditions, disease states or cell types under which a gene is mostly up- or down-regulated. The highest and lowest gene expression states often suggest important functional roles during the corresponding biological processes. None of the above databases can quickly meet this requirement. The Gene Expression Omnibus (GEO) supports gene profile queries^10^, but the results often contain several hundreds to thousands of records. Sifting out meaningful results is often difficult and time-consuming. Expression Atlas is a recently developed database for querying differential GEPs under various experimental factors (i.e., the conditions under study) for multiple organisms^11^. However, both GEO and Expression Atlas are not immune-cell-orientated and neither allow quantitative gene expression heterogeneity and plasticity measurements or marker gene evaluation.

We present a new immunological database, ImmuSort, which provides a global view of GEP under various experiment conditions in a cell-specific pattern. We use gene plasticity (GPL) to describe the expressional heterogeneity of a gene under various experimental conditions. We define it as the change in the expression of a gene in response to various environmental or genetic influences. ImmuSort can retrieve cell states or experimental conditions based on gene expression intensities, which we call electronic sorting. The database offers other functions based on GEP comparisons, such as the evaluation of biomarkers of immune cells and their subsets, identification of cell-specific and/or disease-associated genes or transcripts, comparison of gene expression in different transcript variants and quality evaluation of probe sets in a probe-sequence-independent manner. These features all distinguish ImmuSort from other GEP databases.

## Results

### Design and data statistics

The Affymetrix human genome U133 plus 2.0 and mouse genome 430 2.0 arrays from the GEO database were chosen because these two platforms are currently the most popular arrays and comprise the largest human and mouse study sample sizes, respectively. A quality control analysis resulted in the rejection of about 12% of the original human and 16% of the original mouse samples. The remaining sample for gene expression analysis included 10,422 human samples (GEO samples (GSMs) or microarrays) comprising 433 GEO series (GSEs) and 3,929 mouse samples comprising 455 GSEs. The human and mouse samples were categorised into 31 and 17 groups, respectively, based on cell types, tissue/cell sources, developmental stages and disease states (Table 1, Supplemental Tables S1 and S2).

The human cell types in ImmuSort include T cells, B cells, plasma cells, natural killer (NK) cells, peripheral blood lymphocytes (PBLs), monocytes, macrophages, dendritic cells (DCs), polymorphonuclear leukocytes (PMNs) or neutrophils, mononuclear cells and haematopoietic stem cells (HSCs) (Table 1). An important principle during the sample grouping for these cell types was the requirement of a suitable sample size. Immune cells without enough samples, such as NK T cells and mast cells, were excluded. In contrast, some cell types, such as B cells, T cells, DCs, HSCs, macrophages and peripheral blood mononuclear cells (PBMCs), comprised a great number of samples, which generally allowed for a more subtle division. For example, B cells from patients with acute lymphoblastic leukaemia (ALL), patients with chronic lymphoid leukaemia (CLL) and other patients were grouped separately. T cells were grouped into those from CD4^+^ and CD8^+^ T cell mixtures, those from CD4 single positive T cells alone and those from CD8 single positive T cells alone. Mixed T cell samples from ALL patients were also grouped separately. Due to the relatively large sample size, CD4^+^CD25^+^ regulatory T (Treg) cells, which represent an important subset of helper T cells that modulate the immune system^12^, were separated from the CD4^+^ T cells. Data sets describing other CD4^+^ T cell subsets, such as Th1, Th2 and Th17, still await collection. Similarly, PBMCs were divided into seven groups based on several disease states and the remaining PBMCs were grouped together. DCs, macrophages and HSCs were divided into different groups according to their tissue or cell sources (Table 1).

The mouse cell types in ImmuSort include B cells, T cells, NKs, DCs, HSCs, macrophages, splenocytes and thymocytes. Similarly to the human T cell group, the mouse T cell group is also a mixture of CD4^+^ and CD8^+^ T cells. As enough samples were available, lineage negative Sca-1 positive Kit positive cells and B cell precursors were separated from mouse HSCs and B cells, respectively. Although the human and mouse CD4^+^ T cell samples were both large, no suitable grouping factors (such as developmental stages, phenotypes or disease states) underlying the category could be found. Human CD4^+^ T cells were generally isolated from PBMCs. Mouse CD4^+^ T cells were often isolated from the immune organ spleen. If additional samples become available, these cells may be divided into groups such as naïve or memory CD4^+^ T cells.

### Query

**Figure 1:**
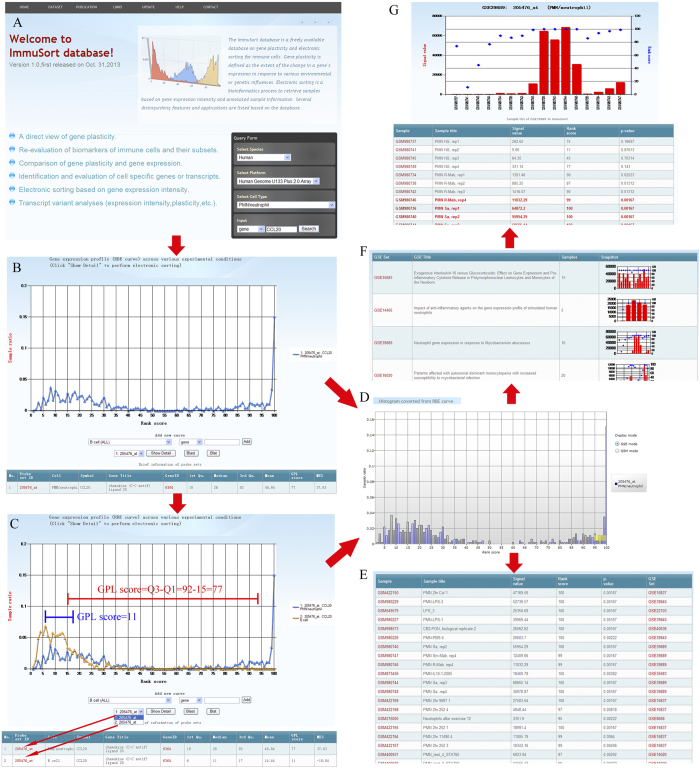
An example illustrating ImmuSort database browsing. (**A**) Search with the gene symbol ‘CCL20’ in human PMNs/neutrophils. (**B**) The query output. The RBE curve indicates the CCL20 GEP and its sample distribution across various experimental conditions. The x-axis represents the percentile rank score that reflects the gene expression intensity. The y-axis represents the sample ratio at an indicated rank score. The button ‘Show Detail’ links to further electronic sorting analysis. The lower table-format panel shows brief information about the queried probe sets. (**C)** A novel RBE curve for CCL20 in B cells is added. The added probe set information is shown in the lower table-format panel in an ordered fashion (see red arrows). (**D**) The GEP is shown in an interactive fashion after finishing the probe set selection and clicking on the ‘Show Detail’ button. Two modes are available to allow users to retrieve or conduct electronic sorting of the cell states (or experiment conditions) that are associated with a bin by clicking it. A bin range is selected by clicking and holding the mouse cursor on a column in the left side and releasing it over another column. In the GSM mode (**E**), all of the cells (GEO samples/GSMs) that are associated with rank scores from 95 to 100 for CCL20 and a brief description of the sample are listed. In the GSE mode (**F**), a list of all of the GSEs containing the cells/GSMs within the selected bin that range from 95 to 100 are shown. The right cell snapshots provide further links to the sample information associated with the GSEs, such as GSE39889 (**G**). In E and G, the signal values, rank score and p values to evaluate the difference between the PM and MM probe values are shown. The items in red (**G**) indicate the samples that are associated with the clicked bin or selected bin range. An online help page on the ImmuSort website also provides operational details.

ImmuSort supports gene symbol or alias (e.g., CCL20 or MIP-3-alpha), Entrez gene ID (e.g., 6364) and probe set ID (e.g., 205476_at) requests as the initial search term. It provides the user with a quick global view of cell-specific gene expression patterns across samples from thousands of experimental conditions (Supplemental Tables S1 and S2). Fig. 1 shows CCL20 used as an example to navigate the database.

In total, 20,283 human and 20,963 mouse genes are currently available for query for each human and mouse cell group. Probe sets without a unique or gene annotation were discarded. A total of 41,477 and 38,544 probe sets for human and mouse, respectively, are available in ImmuSort. Detailed information regarding the probe sets, gene symbols, Entrez Gene IDs and gene aliases are provided in Supplemental Tables S3 (human) and S4 (mouse).

### Features and applications

ImmuSort provides convenient analyses of cell-specific GEPs at the gene or probe set level. It enables the analysis of GPL, evaluation of biomarkers of immune cells and their subsets, gene expression comparisons and other such analyses. Several features and example applications are described below.

### GPL analysis

The rank-based gene expression (RBE) curve can directly illustrate GPL. The GPL score represents the plasticity level. High plasticity indicates that it is common for gene expression to be variable under diverse circumstances and is accompanied by a wide RBE curve. Low plasticity suggests relatively stable expression and is accompanied by a narrow curve. For example, as shown in Fig. 1B and Fig. 1C, CCL20 is highly plastic in PMNs with a GPL score of 77, but weakly plastic in B cells with a GPL score of 11.

GPL has cell- and gene-specificity, which forms the basis of comparison between GEPs. Different RBE curves can be illustrated on the same graph, which allows GEPs from the same gene in different immune cells, or from different genes in the same cell type or group, to be compared. Some applications based on profile comparisons are also available.

### (1) Evaluation of immune cell biomarkers and their subsets

The term ‘marker’ implies homogeneity and stability (although these qualifications are relative), whereas the term ‘plasticity’ represents heterogeneity and variability. Therefore, marker molecules for a cell type should have low plasticity. In contrast, specificity should be observed in a cell type when compared with other cells. ImmuSort provides a direct graphical view of gene expression under varying experimental conditions, allowing marker qualifications to be easily evaluated. Quantitative indices, such as the GPL, average rank score (ARS) and marker evaluation score (MES) can be used in combination to evaluate biomarkers. The GPL score reflects intra-cellular variability. The MES reflects inter-cellular variability. Our unpublished work reveals that a relationship exists between the GPL score and ARS: genes with a small or large ARS often show relatively low plasticity. Genes with a very small or large ARS are therefore likely to have less gene expression variation under diverse conditions, so can be used as marker candidates.

**Figure 2:**
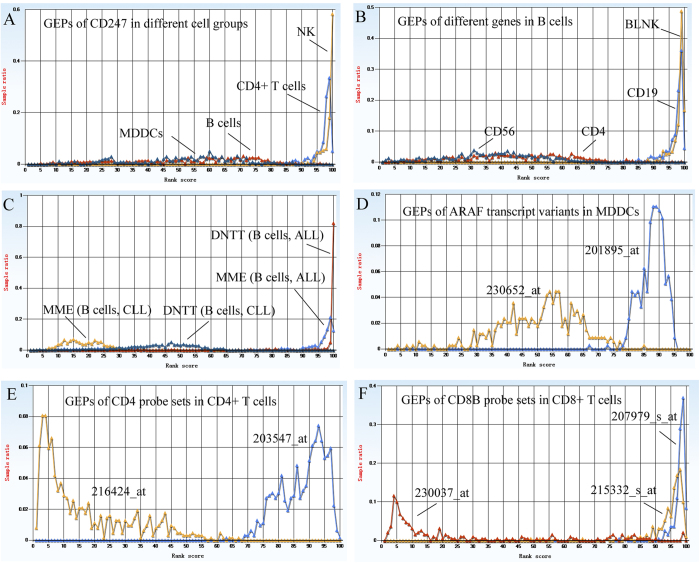
GEP comparison example. (**A**) CD247 is highly expressed and shows low plasticity in CD4^+^ T cells and NK cells. It is weakly expressed and has high plasticity in B cells and MDDCs. (**B**) BLNK and CD19 are strongly expressed and have low plasticity in B cells, whereas CD4 and CD56 are highly plastic and show weak expression in B cells. (**C**) DNTT and MME differential expression in B cells from ALL and CLL patients. (**D**) ARAF transcript variant differential expression in human MDDCs. (**E**) The differential expression of target transcripts that are related to two CD4 probe sets in CD4^+^ T cells reveals a problem with the quality of one of the probe sets. (**F**) The differential expression of target transcripts related to three CD8B probe sets in CD8^+^ T cells reveals a problem with the quality of one of the probe sets.

Low plasticity is insufficient for selecting a marker gene; a gene must also have specificity. The MES is used to describe the potential of a marker molecule because it reflects the variability between cell types. In general, if a gene has low plasticity, the larger the absolute MES value, the better specificity the marker candidate has. For example, CD3Z (CD3-zeta, also known as CD247) is a component of the TCR-CD3 complex that is widely expressed on the surface of T cells. As shown in Fig. 2A, it is highly expressed and has low plasticity in CD4^+^ T cells and NK cells, according to a previous report^13^. However, it tends to have high plasticity and lower expression in other cell types, such as monocyte-derived dendritic cells (MDDCs) and B cells. Human CD4^+^ T cells, MDDCs and B cells have CD247 MES values of 133.25, –29.58 and –32.08, respectively (Supplemental Table S3). The same cells have CD247 ARS values of 96.72, 50.93 and 53.33, respectively (Supplemental Table S5). Similarly, mouse CD247 is highly expressed in CD4^+^ T cells with a MES of 146.22 and an ARS of 96.16, but is weakly expressed in B cells (MES = −43.81, ARS = 38.14) and bone-marrow-derived macrophages (MES = −107.21, ARS  = 30.40) (Supplemental Tables S4 and S6).

As shown in Fig. 2B, CD19 and B-cell linker (BLNK) are highly expressed in B cells under various experimental conditions, whereas CD4 and CD56 are highly plastic and weakly expressed in B cells in general. CD19 is a well-known B cell marker, whereas CD4 and CD56 are key markers of CD4^+^ T cells and NK cells, respectively. BLNK is an essential component of the BCR signalling pathways and plays a critical role in B cell development and function^14^.

The complete MESs and ARSs of the 20,283 human and 20,963 mouse genes are listed in Supplemental Tables S3 and S5 (human) and S4 and S6 (mouse), respectively. The combination of MES and ARS will no doubt contribute to the discovery of novel marker genes. Potential differentially expressed genes can also be identified through these Supplemental Tables and used for further analyses, such as experimental validation and functional enrichment analysis.

### (2) Identification of cell-specific and/or disease-associated genes or transcripts

Cell-specific genes or transcripts indicate that they are widely expressed under various conditions in a cell-type-specific manner. As marker molecules can be effectively evaluated, cell-specific genes or transcripts can also be identified via the database using the MES and ARS. For example, MDDCs highly express genes such as *CD209* (MES = 190.74, ARS = 95.67), transforming growth factor-beta-induced protein (*TGFBI*, MES = 138.49, ARS = 99.26), chemokine C-C motif ligand 22 (*CCL22*, MES = 178.38, ARS = 95.16), transcription factor EC (*TFEC*, MES = 165.99, ARS = 97.52), *CD86* (MES = 182.02, ARS = 98.81) and phospholipase A2, group VII (*PLA2G7*, MES = 166.16, ARS = 97.99) (Supplemental Table S3 and S5). These are all potential marker molecules.

Disease-associated cell states are also provided in ImmuSort, such as B cells from ALL and CLL patients, and can be used to identify disease-associated genes or transcripts. For example, our data show that both membrane metallo-endopeptidase (MME or CD10) and DNA nucleotidylexotransferase (DNTT, also known as terminal deoxynucleotidyl transferase) have distinctively high expression in B cells from ALL patients, but low expression in B cells from CLL patients (Fig. 2C). Indeed, both MME and DNTT are widely used biomarkers for ALL^15^,^16^,^17^. A further comparison of the expression patterns for these two markers in the B cells from ALL patients reveals that DNTT is a relatively more stable marker based on either the expressional intensity or sample proportion because the DNTT peak is higher than the MME peak (Fig. 2C). The ARS and MES of DNTT in the B cells from the ALL patients are 97.3 and 163.13, respectively. The corresponding values for MME are 84.24 (ARS) and 105.26 (MES) (Supplemental Table S3 and S5).

### (3) Transcript variant GEP comparison

For Affymetrix arrays, such as the human genome U133 plus 2.0 and mouse genome 430 2.0 arrays, one gene often corresponds to multiple probe sets. These probe sets may reflect different transcript variants. Thus, the expression profiles of these variants can be directly illustrated and compared. For example, human serine/threonine-protein kinase A-Raf (*ARAF*) has two probe sets, 201895_at and 230652_at. The former corresponds to ARAF transcript variants 1 (NM_001654) and 2 (NM_001256196). The latter corresponds to ARAF transcript variant 3 (NM_001256197), which results from an alternative intronic polyadenylation site^18^. As shown in Fig. 2D, the transcript variant 3 expression level is low in MDDCs under certain experimental conditions.

### (4) Probe set quality evaluation based on GEP

Probe set quality evaluation is usually performed using sequence analysis. Poorly designed probes will produce no or low hybridisation signals. ImmuSort allows the probe quality to be evaluated independently of the probe sequence. For example, CD4 has two probe sets. The transcript related to probe set 203547_at is highly expressed in CD4^+^ T cells. However, the expression profile from the other probe set, 216424_at, is more unstable (as reflected by the GPL score) and is expressed at a low level (Fig. 2E), calling into question the quality of the probe. In fact, the 216424_at probe set does not match a true transcript, but instead matches an untranscribed intronic region (Supplementary Fig. S1). Similarly, CD8B, a component of the CD8 heterodimer, has three probe sets. These include 230037_at, which corresponds to the opposite strand of the CD8B transcript and produces no specific hybrid signals, and 215332_s_at and 207979_s_at, which both produce strong signals in CD8^+^ T cells (Fig. 2F). The target transcripts of 207979_s_at include M1 and M2 transcript variants of the CD8 molecule. Those of 215332_s_at are associated with M3 and M4 transcript variants. Across all CD8^+^ T cells, the M1 variant mRNA levels are the most predominant^19^.

## Electronic sorting of cell states based on gene expression intensity

Cell states corresponding to very high or low gene expression intensities often indicate that a gene has a role in the cellular functions and processes for that cell state. ImmuSort comprises thousands of experimental conditions (Supplemental Tables S1 and S2). These conditions are heterogeneous across sample sources, such as different individuals with unperturbed, perturbed or disease states, and experimental treatments, such as the stimuli and infections that occur during *in vitro* experiments. ImmuSort can guide users to trace experimental conditions based on gene expression intensity, which is called electronic sorting. We define electronic sorting as a bioinformatics process to retrieve cell states or experimental conditions based on gene expression intensity and annotated sample information. ImmuSort provides an easy way to sort cells based on rank scores, which are calculated from gene expression data. The sorted cells, via the rank scores, represent a set of experiments, the cells of which may be derived from normal individuals or patients with certain diseases, or from specific treatments during *in vitro* experiments. Electronic sorting may hint at functional clues for a queried gene.

For example, Fig. 1B shows two peaks in the human CCL20 RBE curve in PMNs/neutrophils. The right peak indicates cell states or experimental conditions with high CCL20 expression levels. Similar peaks can be observed in the CCL20 RBE curves in other myeloid cells, including macrophages, monocytes and DCs. Further electronic sorting of these cell states with high CCL20 expression levels reveals that CCL20 expression is substantially induced by inflammatory stimuli, such as lipopolysaccharides and *Mycobacterium abscessus* strains, which is supported by a large number of GSEs (Supplemental Table S7). CCL20-bright myeloid cells therefore may represent an important functional state during inflammation.

**Figure 3:**
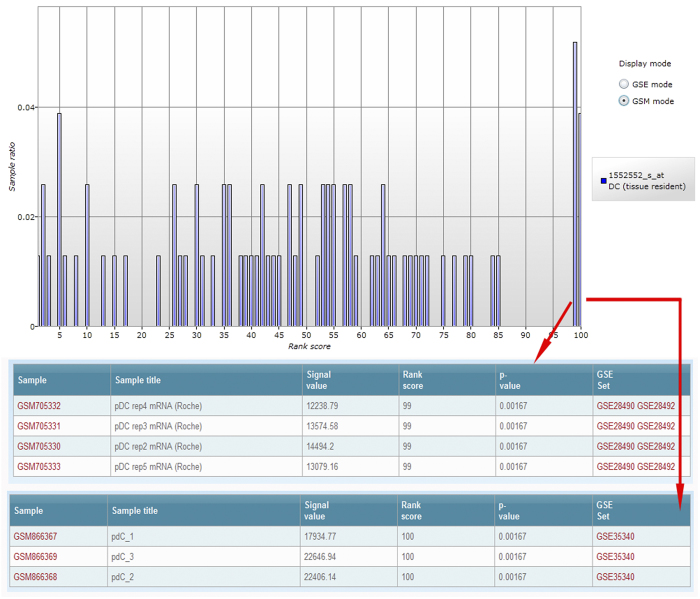
Electronic sorting of DCs with CLEC4C-bright expression. Top histogram indicates the expression profile of human CLEC4C in tissue resident DCs. Middle and bottom panels show cells (or GSM samples) with rank scores of 99 and 100, respectively.

Another example is electronic sorting of cell states with high human C-type lectin domain family 4, member C (CLEC4C, also known as blood dendritic cell antigen 2 (BDCA2) or CD303) expression levels. Fig. 3 shows that BDCA2 is greatly up-regulated in plasmacytoid DCs. However, no rank scores larger than 70 are observed in MDDCs (Supplementary Fig. S2). BDCA2 is a type II C-type lectin receptor and is a highly specific plasmacytoid DC marker^20^,^21^.

Multiple genes cannot be simultaneously queried for electronic sorting in the current version of ImmuSort. However, gene expression data can be downloaded via probe set links in the RBE curve results page, allowing cell states to be systematically traced before electronic sorting. Additionally, downloaded gene expression data can be used for other analyses, such as co-expression analysis in a cell-specific pattern.

## Discussion

An immune cell database was successfully built in this study. To our knowledge, this is the first database describing GPL and electronic sorting (two major distinctive characteristics of the ImmuSort database). The database gives an overview of global cell-specific GEPs in whole cell populations derived under different experiment conditions. Wet experiments usually use over-expression/knock-in, knock-down, knock-out and induction of gene expression to study gene function. Very high or low/no expressional levels of a gene under various pathophysiologic states often indicate potential gene behaviour during the corresponding pathophysiologic processes. The GPL analysis and electronic sorting that are provided by ImmuSort allow users to easily discover under what treatment conditions or disease states, or in what cell types, a gene shows the highest or lowest expression. This is greatly superior to GEO profile analysis because no effective measures are currently available to perform such functions across hundreds and thousands of GEO data sets. However, the GEO database does allow the identification of differentially expressed genes within the same GSE.

Expressional heterogeneity in different cells and plasticity under different experimental conditions provide the basis for GEP comparisons and electronic sorting. GEP comparisons can be used for applications such as biomarker evaluation, cell-specific and/or disease-associated gene or transcript identification, gene expression intensity comparison between different transcript variants and probe set quality evaluation. Electronic sorting allows associations between cell phenotypes and experimental conditions to be traced. It can also help in the discovery of novel marker molecules and immune cell subpopulations.

We evaluated detection calls (present/P, absent/A, marginal/M) based on rank scores. The detection call evaluations in all immune cells revealed that ≥99% of the probe sets were identified as absent if the rank scores were less than 25 for human or 21 for mouse (Supplemental Table S8, Supplemental Fig. S3), suggesting that gene expression was not detected. As microarrays are insensitive to genes with low expression levels, a rank score of 25 (human) or 21 (mouse) can be used as a cut-off for no or undetectable expression, indicating that the signals with lower rank scores may be due to technical noise during DNA hybridisation. If a probe set has a small ARS, it is likely to have very low or no expression under general experimental conditions.

Some cell groups (e.g., PBMCs, PBLs and splenocytes) in ImmuSort are obviously heterogeneous populations. For example, the PBMCs that are widely used in experimental studies are composed of many cell types, including T cells, B cells, NKs, monocytes and a very small portion of DCs. Therefore, the high or low expression states observed during electronic sorting may be due to either changes in gene regulation (or expressional plasticity) within a given cell type or changes in the relative abundance of different cell types. Electronic sorting does not resolve the contribution of different cell types to the global gene expression level in a mixed cell population. Expression deconvolution methods, such as CellMix^22^, can address this problem to some extent. However, computational deconvolution relies on marker gene expression of multiple cell types. We have established effective methods to evaluate quantitative biomarkers for human and mouse immune cells. This will no doubt contribute to deconvolution analysis.

GPL analysis has value for marker evaluation and prediction. Marker evaluation generally cannot be performed in many laboratories due to limited experimental environments and treatment conditions. As the current samples are from different treatment conditions, environments, individuals and cell states, our database provides an effective and feasible solution for bioinformatics-based evaluation and marker molecule prediction in immune cell subsets. For example, our unpublished work suggests that highly plastic genes in a cell type are more suitable as markers to label more subtle populations in cell types. A bioinformatics-based pre-evaluation of candidate markers can effectively improve experiments by providing better marker candidates compared with a random blind approach, saving time and money.

Each probe set in the Affymetrix 3’ expression array usually contains 11–20 probe pairs, each consisting of a PM and an MM oligonucleotide. Although BLAST and BLAT^23^ tools are integrated in ImmuSort for quality probe set analysis, potential cross-hybridisation may still result from one or more probes within a probe set. We recommend that users make a detailed investigation of probe specificity using other tools, such as PLANdbAffy^24^, ADAPT^25^ and X:Map^26^, when an expression profile raises doubts, although probe sets without any annotation or annotated with different targets at the gene level have already been excluded.

With the accumulation of vast high-throughput data from array and next-generation sequencing studies in the GEO^27^ or ArrayExpress^28^ databases, new strategies for data integration are urgently needed. We used the strategy that expressional patterns are reflected through sample distribution based on rank scores. This strategy is also suitable for the analysis of other data, such as microRNA, other non-coding RNA and or mRNA derived from non-immune cells, although it is highly dependent on an adequate sample size. Tissue from different conditions, such as developmental stages and disease states, can also be considered. The increasingly large volumes of accumulated cellular data will facilitate cell-specific studies, such as cell-specific co-expression analysis of immune cells^29^.

An important challenge is categorising heterogeneous cells during cell-specific analyses. The development of cell ontology^30^,^31^ will no doubt contribute to cell-specific studies. However, despite the large amount of public transcriptome data from immune cells, these data are largely restricted to the main immune cell categories, such as CD4^+^ T cells, B cells, monocytes and macrophages. Data sets describing further subsets, such as Th1, Th2 and Th17 cells, have not yet been collected. We believe that as time passes and publicly available data sets accumulate, more grouping factors (such as developmental stages, phenotypes, marker molecules and disease states) can be used for more subtle divisions of the current cell types.

In conclusion, this study successfully developed a web-based platform for integrated immunological transcriptomic data analysis. We plan to integrate more data sets from other GEO platforms and organisms with abundant accumulated data in future versions. In the current version, electronic sorting is based on one GEP. In the next version, we are considering performing electronic sorting based on multiple genes, called multi-dimensional electronic sorting.

## Methods

### Data set and microarray analysis

All of the data related to the Affymetrix human genome U133 plus 2.0 (platform no. GPL570) and mouse genome 430 2.0 (platform no. GPL1261) arrays were downloaded from the GEO database (http://www.ncbi.nlm.nih.gov/geo/)^27^. Previously described methods for data processing and microarray analysis were used^29^. The sample information, such as the sample titles, submission dates, sources and characteristics, was recorded in the downloaded SOFT format files. Search terms related to immune cells, such as ‘T cell’, ‘Th1’, ‘Th2’, ‘Th17’, ‘regulatory T cell’, ‘Treg’, ‘B cell’, ‘natural killer’, ‘NK cell’, ‘plasma cell’, ‘monocyte’, ‘macrophage’, ‘dendritic cell’, ‘neutrophil’, ‘polymorphonuclear leukocyte’, ‘mast cell’, ‘PBMC’, ‘mononuclear cell’, ‘peripheral blood lymphocyte’, ‘hematopoietic stem cell’, ‘haematopoietic stem cell’, ‘HSC’, ‘splenocyte’ and ‘thymocyte’, were used to make an initial evaluation of the samples and their sizes and distributions. To avoid sample leakage, some markers (such as ‘CD4’, ‘CD8’, ‘CD19’, ‘CD56’ and ‘CD34’) and cell name synonyms (such as ‘PBL’ and ‘DC’), were also used for text mining. The results were manually confirmed to avoid false hits. To categorise the immune cells based on experimental factors (such as disease states and tissue sources) and to exclude immune cell leakage, all of the downloaded sample information (not only that pertaining to immune cells) was exported to Microsoft Excel, and the data were scanned by eye. The final cell grouping was therefore performed manually. The data set was last updated on 13 November 2014.

### Affymetrix array quality control

Quality control included a global quality control analysis with the BioConductor package ‘simpleaffy’^32^ and the requirement of gene expression of selected marker genes for each cell type, as described previously in the ImmuCo database^29^. For additional human cell types in ImmuSort, the following quality control markers were used: Tregs: CD4 (203547_at) and CD25 (211269_s_at); PBLs: CD3 (213539_at, 205456_at, 206804_at, 210031_at), CD19 (206398_s_at) and CD56 (212843_at); Jurkat cell lines: CD3 (213539_at, 205456_at, 206804_at, 210031_at); and THP-1 cell lines: CD11b (205786_s_at). For additional mouse cell types in ImmuSort, the following quality control markers were used: B cell precursor: CD19 (1450570_a_at); Lineage negative Sca-1 positive Kit positive haematopoietic stem cells: Ly6e (1453304_s_at) and c-kit (1452514_a_at); and NK cells: NKp46 (1422089_at). For mouse splenocytes, the NK cell marker NKp46 (1422089_at) was considered in addition to CD3 (1426396_at, 1422828_at, 1422105_at, 1419178_at) and CD19 (1450570_a_at).

### Rank scores and RBE curves

All of the probe sets within each array were ranked in ascending order based on their signal intensities. The ranks ranged from 1 to 54,675 for the human genome U133 plus 2.0 arrays and from 1 to 45,101 for the mouse genome 430 2.0 arrays, because there were 54,675 and 45,101 probe sets in each human and mouse array, respectively. The rank order within each array was divided into approximately 100 equal groups. Each group contained 546 or 547 probe sets for human or 451 probe sets for mouse. One hundred rank scores were successively assigned to each group. The probe sets within the same group obtained the same rank scores. The rank scores were ‘1’ and ‘100’ for the lowest and highest 1% of probe sets based on their expressional signal values. The rank scores for the middle portion of the probe sets were set from 2 to 99, in turns. The probe set ARS was the mean of all of the rank scores of the probe set in all of the cell group samples.

Each cell group contained many arrays or GSMs (see Table 1). Each probe set within an array had a rank score. All of the rank scores at the probe set level from all of the arrays within the same cell group constituted a distribution. We used RBE curves to describe these distributions. The x-axis represented the rank score, ranging from 1 to 100. The y-axis represented the percentile ratio for each rank score. For example, if a cell group comprised 500 samples (or arrays) and if there were 100 samples with a rank score equal to 99 for the probe set 1007_s_at, the ratio was 100/500 = 0.2. Therefore, the accumulated square or ratio covered by the RBE curve was equal to 1.

### GPL and MES

The interquartile range, which is the difference between the 1st quartile (25th percentile, 1st Qu, Q1) and 3rd quartile (75th percentile, 3rd Qu, Q3) of gene rank scores at the probe set level across all of the samples within the same cell group, was used as the GPL measurement score.

To facilitate biomarker evaluation, we introduced the MES to quantitatively describe how good a marker was. A background matrix, *M*_*bj*_, was produced, in which *b* represents all of the rank scores of a probe set *j* (therefore, *j* ranged from 1 to 54,675 or 45,101 for human and mouse, respectively) across all cell groups. The rank scores of a probe set across all of the samples within each cell group constituted another matrix, *M*_*ij*_, in which *i* represents the cell group (therefore, *i* ranged from 1 to 31 or 17 for human and mouse, respectively), and *j* represents the probe set. We used the Wilcoxon rank sum test to calculate the p value between *M*_*bj*_ and *M*_*ij*_. The MES was the absolute value of the base 10 logarithm of the p value. If the matrix *M*_*ij*_ median was greater than or equal to that of the corresponding background matrix, the MES was recorded as a positive value, otherwise it was recorded as a negative value. When the p value was zero or the rank scores of a probe set across all of the samples were the same (211943_x_at, 212284_x_at and 212869_x_at for human; only 1454859_a_at for mouse), the MES was recorded as ‘NA’.

The R ( http://www.r-project.org/) language and software environment were used for statistical computing.

### Database architecture

The ImmuSort system is based on a browser and server three-tier architecture. It includes a user interface, business logic and data access. This structure reduces dependence between different system modules and simplifies future system upgrades. The backstage database of ImmuSort was developed on MySQL 5.1 with the MyISAM storage engine.

## Author Contributions

PW designed the study, analysed the data and wrote the manuscript. YY designed the database framework and built the website. WH and DM guided the study and revised the manuscript. All of the authors read and approved the final manuscript.

## Additional Information

**How to cite this article**: Wang, P. *et al.* ImmuSort, a database on gene plasticity and electronic sorting for immune cells. *Sci. Rep.* **5**, 10370; doi: 10.1038/srep10370 (2015).

## Supplementary Material

Supplementary Information

Supplementary Table S1

Supplementary Table S2

Supplementary Table S3

Supplementary Table S4

Supplementary Table S5

Supplementary Table S6

## Figures and Tables

**Table 1:** Immune cells and sample sizes in the current version of the ImmuSort database

**Species**	**Cell type**	**Group**	**Sample size**	**GEO series number**	**Note**
Human					
	B cell	B cell (ALL)	314	9	CD19^+^ B cells from patients with acute lymphoblastic leukaemia (ALL)
		B cell (CLL)	767	16	CD19^+^ B cells from patients with chronic lymphoid leukaemia (CLL)
		B cell	464	45	The rest of the CD19^+^ B cells
	T cell	CD4^+^ T cell	619	55	
		CD8^+^ T cell	189	29	
		T cell	231	30	Mixture of CD4^+^ and CD8^+^ T cells
		T cell (ALL)	230	3	T cells from patients with ALL
		Treg	72	10	Regulatory T cells (Tregs)
		Jurkat	131	22	Acute T cell leukaemia cell line
	NK	NK	149	15	Natural killer cells (NKs)
	PBL	PBL	175	3	Peripheral blood lymphocytes (PBLs)
	Monocyte	Monocyte	462	48	
		THP-1	164	26	Acute monocytic leukaemia cell line
	DC	DC (monocyte-derived dendritic cell, MDDC)	334	25	Monocyte-derived dendritic cells (MDDCs)
		DC (tissue resident)	77	9	Dendritic cells (DCs) from cord blood, peripheral blood and skin, among others. Plasmacytoid DCs are also included in this group.
	Macrophage	Monocyte-derived macrophage (MDM)	253	23	
		Macrophage (lung)	132	7	Alveolar macrophages
	Haematopoietic stem cell	Haematopoietic stem cell (blood)	198	24	CD34^+^ cells from cord blood or peripheral blood
		Haematopoietic stem cell (MDS,bone marrow)	195	3	CD34^+^ cells from the bone marrow of patients with myelodysplastic syndromes (MDS)
		Haematopoietic stem cell (bone marrow)	97	12	CD34^+^ cells from the bone marrow of individuals without MDS
	Mononuclear cell	Bone marrow mononuclear cell (BMMC)	791	11	Bone marrow mononuclear cells (BMMCs) from patients with acute myeloid leukaemia (AML)
		PBMC (breast cancer)	87	2	Peripheral blood mononuclear cells (PBMCs) from patients with breast cancer
		PBMC (CLL)	77	2	PBMCs from patients with CLL
		PBMC (COPD)	94	1	PBMCs from patients with chronic obstructive pulmonary disease (COPD)
		PBMC (JIA)	278	11	PBMCs from patients with juvenile idiopathic arthritis (JIA)
		PBMC (transplant)	150	3	PBMCs from recipients after kidney or liver transplant
		PBMC (MS)	250	2	PBMCs from patients with multiple sclerosis (MS)
		PBMC (miscellaneous)	650	40	PBMCs from miscellaneous and other experimental conditions
		PBMC (normal)	564	39	PBMCs from healthy individuals
	Plasma cell	Plasma cell	1771	18	Mainly from patients with multiple myeloma
	Neutrophil	PMN/neutrophil	457	21	Polymorphonuclear leukocytes (PMNs), neutrophils
					
Mouse					
	B cell	B cell	404	60	CD19^+^ B cells
		B cell precursor	149	20	Including pre-B, pro-B and other B cell precursors
	T cell	CD4^+^ T cell	652	107	
		CD8^+^ T cell	318	41	
		T cell	110	22	Mixture of CD4^+^ and CD8^+^ T cells
		Treg	176	42	
		Thymocyte	267	24	Thymocytes are white blood cells situated in the thymus. They are generally T cells with distinct maturational stages based on the expression of the CD4 and CD8 cell surface markers.
	NK	NK	85	20	
	DC	DC (bone marrow)	147	20	DCs from bone marrow
		DC (spleen)	135	23	DCs from the spleen
		DC (other)	130	12	Primary DCs from other tissue
	Haematopoietic stem cell	Haematopoietic stem cell (LSK)	249	43	CD34^+^ haematopoietic stem cells
		Haematopoietic stem cell (LSK)	208	46	Lineage negative Sca-1 positive Kit positive HSCs
	Macrophage	Macrophage (bone marrow)	571	38	Macrophages derived from bone marrow
		Macrophage (peritoneal)	95	15	Macrophages from the peritoneal
		Macrophage (other)	111	20	Macrophages from other tissue
	Splenocyte	Splenocyte	122	8	Splenocytes are a mixture of different white blood cell types situated in the immune organ spleen.
