# Corrigendum: ImmuSort, a database on gene plasticity and electronic sorting for immune cells

**DOI:** 10.1038/srep20996

**Published:** 2016-02-15

**Authors:** Pingzhang Wang, Yehong Yang, Wenling Han, Dalong Ma

Scientific Reports 5: Article number: 10370; 10.1038/srep10370 published online 05192015; updated: 02152016

The original version of this Article contained typographical errors in the ImmuSort database link ‘ http://immusort.bjmu.edu.cn/.’ which was incorrectly given as ‘ http://202.85.212.211/Account/ImmuSort.html.’ These errors have now been corrected in the PDF and HTML versions of the Article.

