# Supplementary material for: ImmuSort, a database on gene plasticity and electronic sorting for immune cells
Source: Sci Rep. 2015 May 19;5:10370. doi: 10.1038/srep10370 (PMC4437374; doi:10.1038/srep10370)
Supplement: Supplementary Information [file srep10370-s1.pdf]

**Supplementary Information**

**ImmuSort, a database on gene plasticity and electronic sorting for immune cells**

Authors: Pingzhang Wang, Yehong Yang, Wenling Han, Dalong Ma

**Inventory of Supplementary Information**

1. **Supplementary Figure S1.** 216424_at matches an untranscribed intronic region of human gene *CD4*.
2. **Supplementary Figure S2.** No rank scores larger than 70 are observed for human gene *BDCA2* in MDDCs.
3. **Supplemental Table S1**. Sample information for a human data set. The sample title, source name, GEO series and sample description are obtained directly from corresponding records in the GEO database. Please see the online Excel format file, Supplemental Table S1.
4. **Supplemental Table S2**. Sample information for a mouse data set. The sample title, source name, GEO series and sample description are obtained directly from corresponding records in the GEO database. Please see the online Excel format file, Supplemental Table S2.
5. **Supplemental Table S3**. MES of human genes. Please see the online Excel format file, Supplemental Table S3.
6. **Supplemental Table S4**. MES of mouse genes. Please see the online Excel format file, Supplemental Table S4.
7. **Supplemental Table S5**. ARS of human genes. Please see the online Excel format file, Supplemental Table S5.
8. **Supplemental Table S6**. ARS of mouse genes. Please see the online Excel format file, Supplemental Table S6.
9. **Supplemental Table S7**. CCL20-bright myeloid cells are associated with inflammation. Parts of GSEs are listed to show that CCL20 can be substantially induced by inflammatory stimuli in myeloid cells.
10. **Supplemental Table S8 and Figure S3**. Distribution of detection calls at every rank score in all human and mouse immune cells.

## 1. Supplementary Figure S1

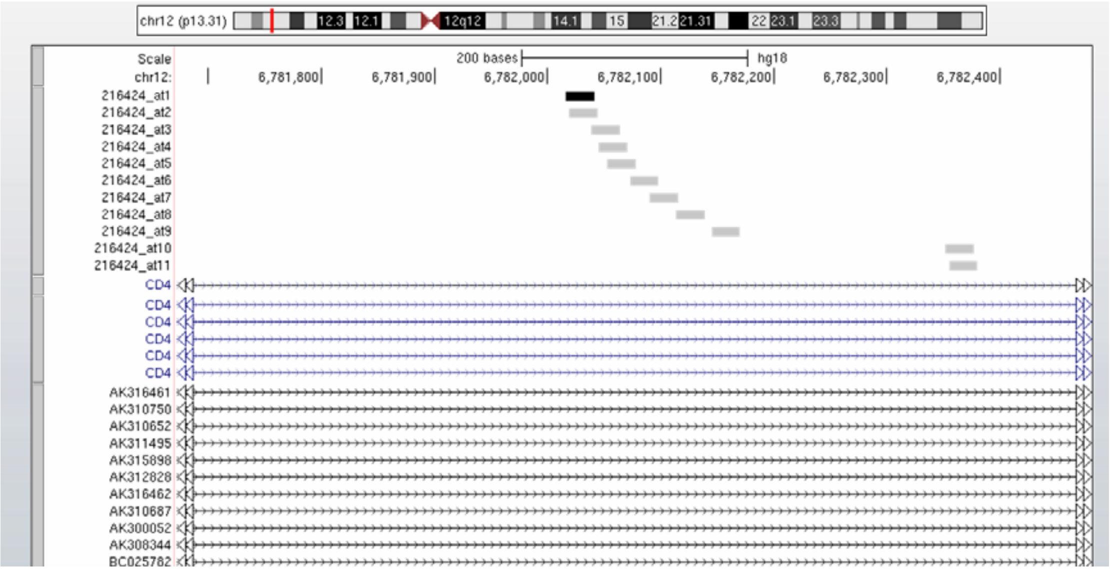

**Figure S1.** 216424_at matches an untranscribed intronic region of human gene *CD4*. The probe set, 216424_at, contains 11 probes from 216424_at1 to 216424_at11. None of the probes match the transcribed region of human *CD4*. The queried result is from the UCSC Genome Browser (http://genome.ucsc.edu/).

## 2. Supplementary Figure S2

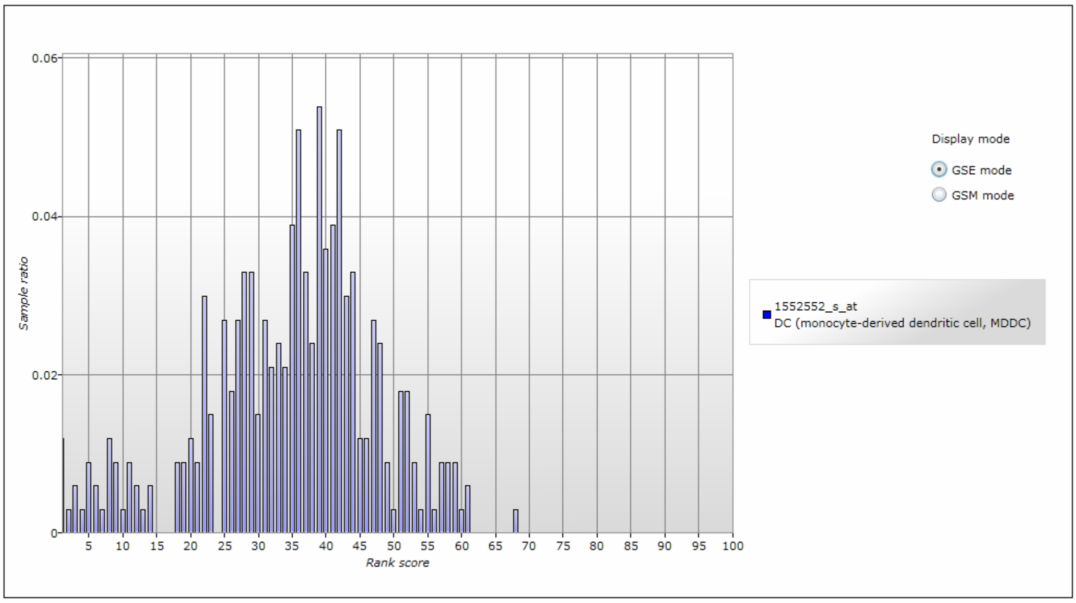


**Figure S2.** No rank scores larger than 70 are observed for human gene *BDCA2* in MDDCs. The histogram is another display mode of RBE curve. The x-axis represents rank scores ranging from 1 to 100. The y-axis represents the percentile ratio at every rank score.

## 3. Supplementary Table S7

**Table S7.** CCL20-bright myeloid cells are associated with inflammation.

| Immune cell | Inflammatory stimulus | GEO series |
|---|---|---|
| PMN/neutrophil | | |
| | S. aureus strains | GSE16837 |
| | mixture of LPS, GM-CSF, TNF-α, fMLP and IL-1β | GSE14465 |
| | LPS, GM-CSF and IFNg | GSE22103 |
| | F. tularensis subsp. | GSE37416 |
| | S. aureus, M. abscessus | GSE39889 |
| | LPS | GSE39840 |
| | E. coli strains | GSE18810 |
| macrophage | | |
| | S. aureus | GSE13670 |
| | Tubercle bacillus extract | GSE11199 |
| | IFN-α | GSE16755 |
| | P. gingivalis (PG), PG-LPS, PG-fimbriae | GSE24897 |
| | LPS | GSE40885 |
| | LPS | GSE8608 |
| monocyte | | |
| | LPS | GSE39840 |
| | TNF-α | GSE38351 |
| | F. novicida, F. tularensis | GSE12108 |
| | LPS | GSE10739 |
| | LPS, IFNg | GSE7307 |
| DC | | |
| | LPS, IFNg | GSE11327 |
| | C. pneumoniae | GSE12806 |
| | LPS | GSE23371 |
| | Newcastle disease virus (NDV) | GSE18791 |
| | Poly(I:C), LPS | GSE10316 |
| | LPS | GSE4984 |
| | H. ducreyi | GSE5547 |
| | A. fumigatus | GSE6965 |

**4. Supplementary Table S8 and Figure S3**.

**Table S8.** Distribution of detection calls at every rank score in all human and mouse immune cells. Detection calls (absent/A, marginal/M and present/P) of all probe sets from 10,422 human and 3,929 mouse samples against each rank score are counted. The corresponding calculated ratios listed below.

| Rank | Human | | | | Mouse | | | |
|---|---|---|---|---|---|---|---|---|
| score | Absent/A | Marginal/M | Present/P | Ratio[A/(A+M+P)] | Absent/A | Marginal/M | Present/P | Ratio[A/(A+M+P)] |
| 1 | 0.010022794 | 3.86085E-08 | 2.98338E-08 | 0.999993171 | 0.010021889 | 3.38597E-08 | 2.82164E-08 | 0.999993806 |
| 2 | 0.009986228 | 2.10592E-08 | 3.33437E-08 | 0.999994552 | 0.009999744 | 1.69298E-08 | 1.69298E-08 | 0.999996614 |
| 3 | 0.010004518 | 3.68536E-08 | 1.75493E-08 | 0.999994562 | 0.009999699 | 5.07895E-08 | 2.82164E-08 | 0.999992099 |
| 4 | 0.010004515 | 2.4569E-08 | 3.33437E-08 | 0.999994211 | 0.009999699 | 3.38597E-08 | 4.51463E-08 | 0.999992099 |
| 5 | 0.010004504 | 4.21184E-08 | 2.6324E-08 | 0.999993159 | 0.00999966 | 6.77194E-08 | 5.07895E-08 | 0.999988149 |
| 6 | 0.009986149 | 6.14226E-08 | 7.19522E-08 | 0.999986644 | 0.009999451 | 1.24152E-07 | 2.03158E-07 | 0.999967268 |
| 7 | 0.010004392 | 9.30114E-08 | 8.77466E-08 | 0.999981932 | 0.00999918 | 2.48304E-07 | 3.49884E-07 | 0.99994018 |
| 8 | 0.010004314 | 1.10561E-07 | 1.47414E-07 | 0.999974214 | 0.009998514 | 5.07895E-07 | 7.562E-07 | 0.999873588 |
| 9 | 0.010004193 | 1.91288E-07 | 1.87778E-07 | 0.999962111 | 0.00999747 | 8.97282E-07 | 1.41082E-06 | 0.999769185 |
| 10 | 0.009985672 | 2.73769E-07 | 3.36947E-07 | 0.999938844 | 0.009996025 | 1.46161E-06 | 2.29117E-06 | 0.999624713 |
| 11 | 0.010003649 | 3.9135E-07 | 5.31744E-07 | 0.999907733 | 0.009993926 | 2.07108E-06 | 3.781E-06 | 0.999414779 |
| 12 | 0.010003125 | 6.21246E-07 | 8.26573E-07 | 0.999855284 | 0.009990348 | 3.56655E-06 | 5.86337E-06 | 0.999056987 |
| 13 | 0.0100023 | 1.00382E-06 | 1.26882E-06 | 0.99977284 | 0.00998637 | 4.98866E-06 | 8.41978E-06 | 0.998659126 |
| 14 | 0.009982889 | 1.42851E-06 | 1.96552E-06 | 0.99966013 | 0.009980529 | 6.87352E-06 | 1.23757E-05 | 0.998075034 |
| 15 | 0.009999536 | 2.09539E-06 | 2.94126E-06 | 0.999496565 | 0.009972256 | 1.00112E-05 | 1.75111E-05 | 0.99724771 |
| 16 | 0.009997498 | 2.91494E-06 | 4.15919E-06 | 0.99929291 | 0.009961641 | 1.3826E-05 | 2.43113E-05 | 0.996186185 |
| 17 | 0.009993653 | 4.47683E-06 | 6.44235E-06 | 0.998908581 | 0.009949293 | 1.77538E-05 | 3.2731E-05 | 0.994951407 |
| 18 | 0.009970657 | 6.12647E-06 | 9.49944E-06 | 0.998435263 | 0.00993356 | 2.33406E-05 | 4.28777E-05 | 0.993378025 |
| 19 | 0.009982027 | 8.96243E-06 | 1.35832E-05 | 0.99774647 | 0.009911591 | 3.11735E-05 | 5.70141E-05 | 0.991181047 |

| | | | | | | | | |
|---|---|---|---|---|---|---|---|---|
| 20 | 0.009973528 | 1.20423E-05 | 1.90024E-05 | 0.996896945 | 0.009886676 | 3.95594E-05 | 7.35433E-05 | 0.988689482 |
| 21 | 0.00996148 | 1.68666E-05 | 2.62257E-05 | 0.995692735 | 0.009855976 | 5.03832E-05 | 9.34189E-05 | 0.985619468 |
| 22 | 0.009927433 | 2.23455E-05 | 3.65043E-05 | 0.994106929 | 0.009816501 | 6.37296E-05 | 0.000119547 | 0.981671905 |
| 23 | 0.00992602 | 2.93091E-05 | 4.92434E-05 | 0.992148342 | 0.009774284 | 7.74258E-05 | 0.000148068 | 0.977450071 |
| 24 | 0.009901003 | 3.91139E-05 | 6.44551E-05 | 0.98964783 | 0.009718511 | 9.6946E-05 | 0.000184321 | 0.971872691 |
| 25 | 0.009870427 | 4.91416E-05 | 8.50036E-05 | 0.986591611 | 0.009658281 | 0.000114976 | 0.000226521 | 0.965849482 |
| 26 | 0.009815054 | 6.25721E-05 | 0.000108657 | 0.982853614 | 0.009592836 | 0.000135811 | 0.000271132 | 0.959304822 |
| 27 | 0.009789281 | 7.79172E-05 | 0.000137374 | 0.978480692 | 0.009515822 | 0.000160647 | 0.000323309 | 0.951603264 |
| 28 | 0.009739959 | 9.38292E-05 | 0.000170785 | 0.973550712 | 0.009436957 | 0.000182854 | 0.000379968 | 0.943716602 |
| 29 | 0.00966231 | 0.00011333 | 0.000210643 | 0.967558237 | 0.009348369 | 0.000210077 | 0.000441333 | 0.93485758 |
| 30 | 0.009618045 | 0.000133431 | 0.000253096 | 0.961364951 | 0.00924973 | 0.000238135 | 0.000511913 | 0.924993468 |
| 31 | 0.009545981 | 0.000156682 | 0.00030191 | 0.954161795 | 0.009146062 | 0.000266521 | 0.000587195 | 0.914626528 |
| 32 | 0.009468667 | 0.00017895 | 0.000356955 | 0.946433978 | 0.009036622 | 0.000293942 | 0.000669214 | 0.903682267 |
| 33 | 0.009368898 | 0.000202126 | 0.000415259 | 0.938176708 | 0.008912594 | 0.000326176 | 0.000761008 | 0.891279186 |
| 34 | 0.009298674 | 0.000227811 | 0.000478087 | 0.929442429 | 0.008792753 | 0.000353997 | 0.000853027 | 0.879294845 |
| 35 | 0.009204354 | 0.000254627 | 0.000545592 | 0.920014686 | 0.008659166 | 0.000385989 | 0.000954623 | 0.86593577 |
| 36 | 0.009102454 | 0.000278995 | 0.000623123 | 0.909829334 | 0.008518998 | 0.000412298 | 0.001068482 | 0.851918674 |
| 37 | 0.008979003 | 0.000307436 | 0.000699844 | 0.899133666 | 0.008371302 | 0.000448331 | 0.001180146 | 0.837148747 |
| 38 | 0.008887625 | 0.000334204 | 0.000782743 | 0.888356335 | 0.008211326 | 0.000478584 | 0.001309868 | 0.821150815 |
| 39 | 0.008769636 | 0.000360942 | 0.000873994 | 0.876562798 | 0.00805192 | 0.000506293 | 0.001441565 | 0.805209881 |
| 40 | 0.008644201 | 0.000390165 | 0.000970207 | 0.864024983 | 0.007882221 | 0.000536704 | 0.001580853 | 0.78823959 |
| 41 | 0.008507893 | 0.000412821 | 0.001065568 | 0.851957995 | 0.007698137 | 0.00056382 | 0.001737821 | 0.769830794 |
| 42 | 0.008391575 | 0.000441163 | 0.001171834 | 0.838773941 | 0.007509082 | 0.000590107 | 0.00190059 | 0.750924813 |
| 43 | 0.008255596 | 0.000467368 | 0.001281609 | 0.825182245 | 0.007311036 | 0.000620372 | 0.00206837 | 0.731119838 |
| 44 | 0.008112321 | 0.00049362 | 0.001398631 | 0.810861358 | 0.007110339 | 0.00064251 | 0.002246929 | 0.711049623 |
| 45 | 0.007948781 | 0.000517921 | 0.001519581 | 0.795969958 | 0.006888174 | 0.000669068 | 0.002442537 | 0.688832655 |

| | | | | | | | | |
|---|---|---|---|---|---|---|---|---|
| 46 | 0.007807776 | 0.00054247 | 0.001654326 | 0.780420724 | 0.006661596 | 0.000693294 | 0.002644888 | 0.666174373 |
| 47 | 0.007646376 | 0.000567062 | 0.001791134 | 0.764288173 | 0.006429866 | 0.000710495 | 0.002859417 | 0.643000848 |
| 48 | 0.007476187 | 0.000589122 | 0.001939264 | 0.747276977 | 0.006186894 | 0.000726539 | 0.003086345 | 0.618703156 |
| 49 | 0.007285362 | 0.000610874 | 0.002090046 | 0.729536983 | 0.005933212 | 0.00074379 | 0.003322776 | 0.593334345 |
| 50 | 0.007119655 | 0.000631168 | 0.00225375 | 0.711640086 | 0.005691504 | 0.000753412 | 0.003577034 | 0.567903855 |
| 51 | 0.006924699 | 0.000653078 | 0.002426795 | 0.692153464 | 0.005414137 | 0.000757447 | 0.003828194 | 0.541425717 |
| 52 | 0.006714269 | 0.000670056 | 0.002601958 | 0.672349208 | 0.005131335 | 0.000767853 | 0.00410059 | 0.513144907 |
| 53 | 0.006520156 | 0.00068705 | 0.002797366 | 0.651717626 | 0.004858968 | 0.000767436 | 0.004373375 | 0.485907564 |
| 54 | 0.00630294 | 0.000703364 | 0.002998269 | 0.63000589 | 0.004575066 | 0.000770184 | 0.004654529 | 0.457516709 |
| 55 | 0.006079611 | 0.000714676 | 0.003210285 | 0.607683192 | 0.004283483 | 0.000765624 | 0.004950671 | 0.428357785 |
| 56 | 0.005838751 | 0.000723648 | 0.003423883 | 0.584677173 | 0.003998136 | 0.000752961 | 0.005248682 | 0.399822458 |
| 57 | 0.005614627 | 0.000734944 | 0.003655001 | 0.561206132 | 0.00371648 | 0.000742673 | 0.005540626 | 0.37165621 |
| 58 | 0.005366055 | 0.000743005 | 0.003895512 | 0.536360294 | 0.003426607 | 0.000721945 | 0.005851226 | 0.342668282 |
| 59 | 0.005118333 | 0.00074639 | 0.00413985 | 0.511599355 | 0.003150955 | 0.00070067 | 0.006148153 | 0.315102493 |
| 60 | 0.004857397 | 0.000746865 | 0.00438202 | 0.48640696 | 0.002876076 | 0.000680794 | 0.006442908 | 0.287614018 |
| 61 | 0.004611084 | 0.000744482 | 0.004649006 | 0.460897651 | 0.002616006 | 0.000651291 | 0.006732481 | 0.261606373 |
| 62 | 0.004355415 | 0.000738003 | 0.004911154 | 0.435342443 | 0.002362673 | 0.000622742 | 0.007014363 | 0.236272552 |
| 63 | 0.004102663 | 0.000732173 | 0.005169737 | 0.410078771 | 0.002118731 | 0.00059088 | 0.007290167 | 0.211877793 |
| 64 | 0.003841324 | 0.000719608 | 0.005425351 | 0.384660021 | 0.001895618 | 0.000558792 | 0.007545368 | 0.189566016 |
| 65 | 0.003594549 | 0.000708224 | 0.0057018 | 0.359290588 | 0.001678312 | 0.000522545 | 0.007798921 | 0.167834946 |
| 66 | 0.003350152 | 0.000691855 | 0.005962565 | 0.334862057 | 0.001487993 | 0.000486067 | 0.008025719 | 0.148802554 |
| 67 | 0.003106118 | 0.000673915 | 0.00622454 | 0.310469837 | 0.001306318 | 0.00044925 | 0.008244209 | 0.130634731 |
| 68 | 0.002867047 | 0.000651832 | 0.006467403 | 0.287098544 | 0.001149238 | 0.000413184 | 0.008437356 | 0.114926306 |
| 69 | 0.002644276 | 0.00062992 | 0.006730376 | 0.264306766 | 0.000999973 | 0.000376238 | 0.008623568 | 0.099999492 |
| 70 | 0.00242873 | 0.000604509 | 0.006971333 | 0.242762024 | 0.000866594 | 0.000342372 | 0.008790812 | 0.086661298 |
| 71 | 0.002219069 | 0.000578432 | 0.007207071 | 0.221805441 | 0.00075168 | 0.000310121 | 0.008937978 | 0.075169627 |

| | | | | | | | | |
|---|---|---|---|---|---|---|---|---|
| 72 | 0.002018933 | 0.000549888 | 0.007417461 | 0.202170599 | 0.000650022 | 0.000277971 | 0.009071786 | 0.065003592 |
| 73 | 0.001833758 | 0.000521541 | 0.007649274 | 0.183291953 | 0.00056044 | 0.000250302 | 0.009189036 | 0.056045247 |
| 74 | 0.001656766 | 0.000492167 | 0.00785564 | 0.165600858 | 0.000475729 | 0.000224196 | 0.009299853 | 0.047573927 |
| 75 | 0.001489989 | 0.000460004 | 0.008036289 | 0.149203608 | 0.000407795 | 0.0001969 | 0.009395084 | 0.040780393 |
| 76 | 0.001335738 | 0.000430392 | 0.008238443 | 0.133512746 | 0.000346435 | 0.000175681 | 0.009477662 | 0.034644316 |
| 77 | 0.001191765 | 0.00040137 | 0.008411437 | 0.119122044 | 0.000293761 | 0.000154163 | 0.009551854 | 0.029376759 |
| 78 | 0.001055814 | 0.000369102 | 0.008579656 | 0.105533155 | 0.000246346 | 0.000135405 | 0.009618027 | 0.024635168 |
| 79 | 0.000934927 | 0.000339925 | 0.00871143 | 0.093621165 | 0.000204823 | 0.000117651 | 0.009677304 | 0.020482748 |
| 80 | 0.000822112 | 0.000311732 | 0.008870729 | 0.082173591 | 0.000169823 | 0.000100072 | 0.009729883 | 0.016982707 |
| 81 | 0.000720861 | 0.000282426 | 0.009001285 | 0.072053142 | 0.000141178 | 8.64946E-05 | 0.009772106 | 0.014118113 |
| 82 | 0.000624384 | 0.000257901 | 0.009122288 | 0.062409816 | 0.000115179 | 7.26234E-05 | 0.009811975 | 0.011518195 |
| 83 | 0.00054005 | 0.000230435 | 0.009215798 | 0.054079213 | 9.42541E-05 | 6.08289E-05 | 0.009844695 | 0.00942562 |
| 84 | 0.000463161 | 0.000206552 | 0.009334859 | 0.046294981 | 7.61561E-05 | 5.07783E-05 | 0.009872844 | 0.007615779 |
| 85 | 0.00039539 | 0.000182571 | 0.009426612 | 0.039520884 | 6.28605E-05 | 4.40966E-05 | 0.009892821 | 0.006286192 |
| 86 | 0.000332107 | 0.000159795 | 0.00951267 | 0.033195494 | 5.12579E-05 | 3.49376E-05 | 0.009913583 | 0.005125907 |
| 87 | 0.000277318 | 0.000139164 | 0.009569801 | 0.02776987 | 3.86226E-05 | 2.95764E-05 | 0.009931579 | 0.003862348 |
| 88 | 0.000230505 | 0.000119623 | 0.009654444 | 0.023039962 | 3.15911E-05 | 2.43451E-05 | 0.009943842 | 0.00315918 |
| 89 | 0.000187504 | 0.000102104 | 0.009714965 | 0.018741819 | 2.45821E-05 | 1.98982E-05 | 0.009955298 | 0.002458268 |
| 90 | 0.000152291 | 8.49703E-05 | 0.009767311 | 0.015222159 | 1.97797E-05 | 1.5773E-05 | 0.009964226 | 0.001978014 |
| 91 | 0.000120685 | 6.99042E-05 | 0.009795694 | 0.012085065 | 1.4475E-05 | 1.26917E-05 | 0.009972612 | 0.001447534 |
| 92 | 9.54226E-05 | 5.66281E-05 | 0.009852522 | 0.009537903 | 1.11963E-05 | 1.02313E-05 | 0.009978351 | 0.001119652 |
| 93 | 7.22014E-05 | 4.51983E-05 | 0.009887173 | 0.007216839 | 8.85431E-06 | 7.75951E-06 | 0.009983164 | 0.000885451 |
| 94 | 5.43432E-05 | 3.46423E-05 | 0.009915587 | 0.005431837 | 6.65907E-06 | 6.0101E-06 | 0.009987109 | 0.000665922 |
| 95 | 3.91437E-05 | 2.52306E-05 | 0.009921908 | 0.003919751 | 4.34533E-06 | 4.27196E-06 | 0.009991161 | 0.000434542 |
| 96 | 2.66381E-05 | 1.75476E-05 | 0.009960387 | 0.002662593 | 3.39161E-06 | 3.20538E-06 | 0.009993181 | 0.000339169 |
| 97 | 1.68631E-05 | 1.17458E-05 | 0.009975964 | 0.001685543 | 2.33068E-06 | 2.42097E-06 | 0.009995027 | 0.000233073 |

| | | | | | | | | |
|---|---|---|---|---|---|---|---|---|
| 98 | 9.18707E-06 | 7.03376E-06 | 0.009988352 | 0.000918287 | 1.60834E-06 | 2.2686E-06 | 0.009995901 | 0.000160837 |
| 99 | 3.71519E-06 | 3.82926E-06 | 0.009978738 | 0.000372029 | 1.0045E-06 | 2.92322E-06 | 0.009995851 | 0.000100453 |
| 100 | 1.83741E-06 | 3.00971E-06 | 0.009999725 | 0.000183657 | 1.80585E-06 | 9.48636E-06 | 0.009966314 | 0.00018099 |

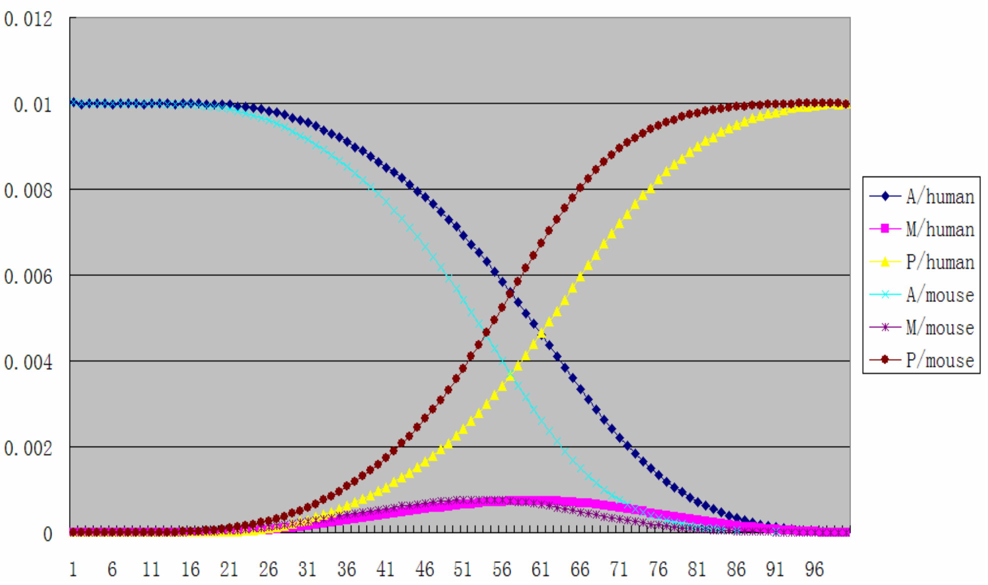


**Figure S3.** Distribution of detection calls at every rank score in all human and mouse immune cells. The x-axis indicates the rank score. The y-axis represents the probe set ratio at each rank score. The values are taken from the columns absent/A, marginal/M and present/P in Table S8.
